# Network topology facilitates internet traffic control in autocracies

**DOI:** 10.1093/pnasnexus/pgae069

**Published:** 2024-02-14

**Authors:** Eda Keremoğlu, Nils B Weidmann, Alexander Gamero-Garrido, Esteban Carisimo, Alberto Dainotti, Alex C Snoeren

**Affiliations:** Department of Politics and Public Administration, University of Konstanz, 78457 Konstanz, Germany; Department of Politics and Public Administration, University of Konstanz, 78457 Konstanz, Germany; Department of Computer Science, University of California, Davis, CA 95616-5270, USA; Department of Computer Science, Northwestern University, Evanston, IL 60208, USA; School of Computer Science, Georgia Institute of Technology, Atlanta, GA 30332, USA; Department of Computer Science and Engineering, University of California San Diego, La Jolla, CA 92093, USA

## Abstract

Recent years have seen an increase in governmental interference in digital communication. Most research on this topic has focused on the application level, studying how content is manipulated or removed on websites, blogs, or social media. However, in order for governments to obtain and maintain control of digital data flows, they need to secure access to the network infrastructure at the level of Internet service providers. In this paper, we study how the network topology of the Internet varies across different political environments, distinguishing between control at the level of individual Internet users (access) and a higher level in the hierarchy of network carriers (transit). Using a novel method to estimate the structure of the Internet from network measurements, we show that in autocratic countries, state-owned (rather than privately owned) providers have a markedly higher degree of control over transit networks. We also show that state-owned Internet providers often provide Internet access abroad, with a clear focus on other autocratic countries. Together, these results suggest that in autocracies, the network infrastructure is organized in a way that is more susceptible to the monitoring and manipulation of Internet data flows by state-owned providers both domestically and abroad.

**Significance Statement**

Most research on autocratic control of the digital information environment has focused on social media and website content. However, information control is more effective when governments can influence how data is transmitted over the underlying network infrastructure. Our study investigates how this infrastructure is set up in autocracies and democracies, and which role government-owned Internet providers play. We find that first, in autocratic countries, government-owned providers retain a much more central role as domestic *transit* carriers, which relay data flows from the country to the global Internet. Second, in many autocracies, Internet *access* is provided by state-owned providers from other autocratic countries, which illustrates international patterns of collaboration between these countries.

## Introduction

On 30 April 2022, several weeks after the Russian invasion of Ukraine, the Kherson district in Eastern Ukraine experienced a complete Internet shutdown. When service returned a day later, Internet traffic was routed through Rostelecom, Russia's main and state-owned Internet provider (1). This incident is only one of the latest in a series of moves to make Russian-occupied areas in Ukraine part of the Russian Internet (2). It illustrates that governmental control over Internet traffic flows remains a fundamental concern in particular for autocratic regimes, presumably to retain opportunities to interfere with digital communication (3).

This governmental interference can happen in different ways (4). The blocking of access to particular websites (5) or even the shutdown of the entire domestic Internet (6, 7) constitute extreme forms of Internet control; more subtle techniques include censorship of content deemed unacceptable (8) or the spread of misinformation (9). For most of these tactics, close collaboration with Internet providers is necessary. Existing research has revealed much about these observable manifestations of governmental interference. So far, however, we know little about whether the underlying Internet topology in autocracies is set up to facilitate governmental incursions in cyberspace. In this article, we use a new dataset on the impact of providers on Internet data streams, which was created with Internet measurement techniques. We leverage these data to study how Internet topology differs between autocracies and democracies. In particular, we focus on the role of state-owned Internet providers, which can be an effective way for governments to influence and control communication flows on the domestic Internet, but also abroad.

Two groups of networks in the Internet infrastructure play a key role in carrying traffic: (i) access providers and (ii) transit and backbone providers. An *access* network connects individuals to the Internet at large, typically in the form of fixed broadband or high-speed cellular networks. *Transit* providers are responsible for connecting customer access networks to the rest of the Internet; some transit providers are rather large and serve as the “core” or backbone of the Internet, including so-called tier-1 providers. While access networks are a critical surveillance point, the structure of transit networks underpinning the modern Internet may be equally consequential in determining the exposure to interference in a country's traffic. Therefore, our focus is not solely on the access networks, but on end-to-end infrastructure. In all these types of networks, governments’ engagement in the Internet service business would give them direct capabilities to observe and tamper with Internet traffic. In fact, state involvement in service provision is frequent, with governments owning majority stakes in domestic Internet providers in 123 countries globally (10). However, what is their influence on Internet traffic?

## Results

In the first analysis, we examine the influence of domestic providers across democracies and autocracies. Providers keep records of users or households, which allows for a simple matching of (possibly suspicious) data traffic to individuals. Therefore, we would expect control over *access* providers to be particularly important for autocratic governments. To measure the influence these access providers have on domestic traffic, we use the share of domestic IP addresses owned by the respective provider (11). We combine these data with a second dataset by Carisimo et al. (10) to identify state-owned providers.

**Fig. 1.**
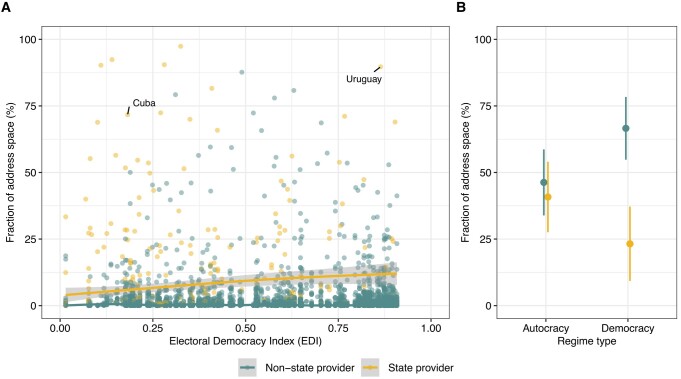
A) Fraction of address space serviced by individual domestic states vs. nonstate providers, for nondemocratic (low values of the electoral democracy index, left) and democratic countries (right). B) Predicted fraction of address space serviced by all domestic state vs. nonstate providers based on regression results in Section 4.1 of supplementary material (model 4). Binary regime type indicator derived from the electoral democracy index (12,).

In Fig. 1A, we plot the relative influence of domestic access providers across different regime types. The plot shows that counter to common expectations, the type of political regime is not necessarily a predictor of the state's monopoly of the address space. For instance, Uruguay and Cuba (which have regimes classified as democracy and autocracy, respectively) both have state-sanctioned monopolies (ANTEL and ETECSA). Regression models controlling for potential confounders confirm this (see Section 4.1 of supplementary material). This analysis shows that there is no difference in the fraction of address space controlled by state and nonstate providers in autocracies while in democracies, state-owned providers are significantly less involved in access provision (Fig. 1 B). In Section 5 of supplementary material, we check that these results are robust to the use of alternative democracy indicators, a reduced sample, and a different assignment of providers to countries.

An alternative, and likely more efficient, way to monitor and interfere with Internet traffic is to control data traffic at the level of *transit* providers. For governments attempting to regulate digital flows of information, controlling the underlying infrastructure as a transit provider can vastly expand the potential to control the functionality and content of online communication. As transit networks are often unaccountable to consumers, they create opportunities for government surveillance and censorship without facing political backlash. Further, transit networks can serve multiple access networks (both fixed and wireless) simultaneously, allowing for the creation of a centralized observation or manipulation point. We measure the degree to which state-owned transit providers influence transit flows using a new dataset compiled by Gamero-Garrido et al. (13). The dataset improves on earlier attempts (14) by quantifying the capabilities of a transit network to observe or selectively tamper with a country's inbound traffic. Our analyses of transit influence comprise a sample of 75 countries. We provide more details on the sample of countries and the data generation and assess possible sampling bias in the Supplementary material.

**Fig. 2.**
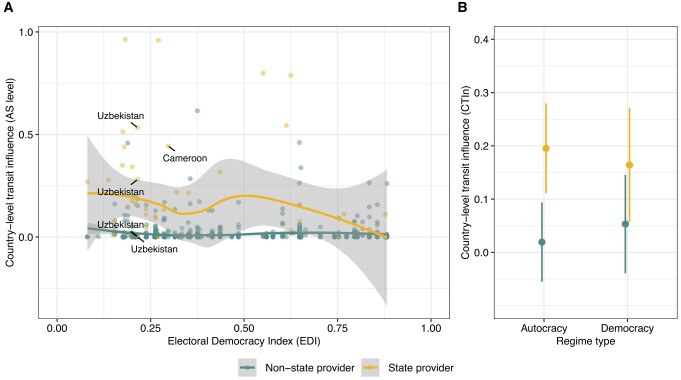
A) Country-level transit influence (CTI) of individual domestic state vs. nonstate providers, for nondemocratic (low values of the electoral democracy index, left) and democratic countries (right). CTI estimates the relative prevalence of a particular network on Internet routes serving a country and ranges from 0 to 1, with higher values denoting higher prevalence on routes that reach a higher number of IP addresses. B) Predicted average country-level transit influence (CTIn) of domestic state vs. nonstate providers in democratic and nondemocratic countries, based on regression results in Section 4.2 of supplementary material (model 4). CTIn is the combined transit influence, ranging from 0 to 1, of the group of all individual state-owned vs. nonstate providers. Binary regime type indicator derived from the electoral democracy index (12). Robustness tests for this analysis are provided in Section 5 of supplementary material.

Figure 2A plots the transit influence of domestic providers across the range of democracies and autocracies. Two autocracies, Cameroon and Uzbekistan, operate highly influential and state-owned transit networks: *Camtel* and *Uzbektelekom*. These operators provide international connectivity to a significant fraction of their respective country's users. In general, compared to other (nonstate) domestic transit providers, the influence of state-owned providers is particularly high in autocracies (panel A, left), while it decreases in the democratic countries in the sample (panel A, right). A statistical analysis with different controls confirms this result (Fig. 2B): In autocracies, state-owned domestic providers have a much higher combined influence on domestic Internet traffic as compared to nonstate providers, a difference we do not see in democracies. These analyses suggest that authoritarian governments maintain a high level of control over the domestic Internet traffic not as access providers, but rather as transit traffic carriers.

Our analysis so far has focused only on the domestic influence of Internet providers. However, many providers operate internationally. While their influence at the transit level is negligible (in our sample, the country-level median transit influence is only 0.0006), in many countries, foreign access providers control a significant share of the Internet access (global average: 25.7%, autocracies: 27.5%, and democracies: 24%). These providers have considerable control over Internet traffic as a form of international influence. We therefore focus on access service provision at the level of individual Internet users in the subsequent analyses.

**Fig. 3.**
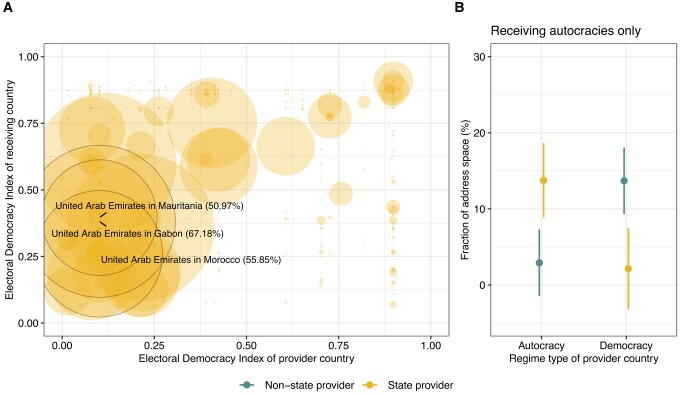
A) Address space serviced by individual state-owned access providers from democratic and nondemocratic countries (*x*-axis), ordered by the level of democracy of the country where the service is provided (*y*-axis). The size of the dots represents the share of address space serviced by individual providers. B) Predicted aggregate address space originated by all state vs. nonstate access providers in other autocracies only. Figure based on regression results in Section 4.3 of supplementary material (model 4). Binary regime type indicator derived from the electoral democracy index (12). Robustness tests in Section 5 of supplementary material.

In Fig. 3A, we visualize the extent to which state-owned providers control access abroad, distinguishing between the different regime types (i) where these providers come from (*x*-axis) and (ii) where they operate (*y*-axis). The plot shows that the operation of state-owned providers from autocratic countries concentrates largely on other autocracies (most of the dots cluster at the left bottom), where these providers are highly influential (thus the larger dots). The United Arab Emirates is a case in point. Three of its state-owned providers operate in Gabon, Mauritania, and Morocco, respectively, where the share of access they control is high, ranging from 50 to 67%. Using again regression analysis to back up these descriptive results (Fig. 3B), we confirm that foreign access providers operating in autocracies are almost exclusively located in other autocratic countries. This shows that the international operation of access providers follows a political logic. State-owned providers from some autocratic countries are highly influential in other autocracies, thus creating clusters of autocratic cooperation in the Internet infrastructure.

For pervasive and effective use of Internet communication, it is necessary that governments maintain control over data flows on the Internet, independently of what service or application they are used for. In this paper, we have documented considerable differences in the topology of the Internet infrastructure across democratic and autocratic countries. Using a new method to estimate the influence of Internet providers on the data traffic between users and the global Internet, we have shown that in particular in autocratic countries, the Internet infrastructure is set up such that domestic state-owned Internet providers retain a much greater level of control over transit Internet traffic. These features of the network topology can vastly expand the opportunities for nondemocratic governments to monitor sensitive information, censor critical content, and disrupt digital communication domestically. Our results also reveal the increasing potential of governmental Internet control across state borders. While democracies and autocracies rely on foreign access providers to a similar extent, we find that many state-owned providers from autocratic countries operate preferably in other autocratic countries, thus creating clusters of technological cooperation between nondemocratic countries.

## Discussion

Our study has important implications for research and information and communication technology policy. First, to gain a better understanding of the political role of the internet, researchers need to take into account the network topology and the different ways in which service providers operate in it. More sophisticated, less observable means of potential influence may work even more to the advantage of governments to avert threats to their rule during liberation struggles (15). Along these lines, existing work on digital interference at the application layer needs to be supplemented with analyses of the underlying network topology, to better understand who can control (and potentially manipulate) the data traffic these applications are based on. Second, while international providers from democratic states need to be aware of their political impact and assess their abilities to influence traffic in nondemocratic states, our results also show that their influence may be limited. As our results have shown, we see close technological cooperation evolve between autocratic countries at the level of Internet service provision. With state-owned autocratic providers operating in other autocratic countries, potentially differing standards of privacy and anonymity can be avoided, thus sustaining autocratic rule through international partnerships.

Nevertheless, our focus on state ownership only addresses one aspect of an autocratic government's potential to control the digital information environment. In addition to the direct means we assess in our study, governments exert considerable control also over private firms that operate under the state's jurisdiction, which provides them with additional, indirect means of interfering with network traffic (16). Focusing on China, research has shown that the political environment is an important factor to consider when market dynamics and the location of firms shape how autocrats exert influence via private domestic firms. Following Ref. (17), this could make customers serviced by those providers still vulnerable to influence. China is not an outlier; requiring nonstate providers to comply with repressive governmental regulations is common practice also in many other autocracies, such as Russia, Saudi Arabia, or the UAE (16). As a result, our analysis that focuses on ownership as a direct means of control produces a conservative estimate of the extent to which states can interfere with digital communication, which in reality may be more severe.

## Supplementary Material

pgae069_Supplementary_Data

## Data Availability

The data and R code required to replicate the results of the statistical analyses are available at https://doi.org/10.7910/DVN/59BX4J
