# Supplementary material for: Network topology facilitates internet traffic control in autocracies
Source: PNAS Nexus. 2024 Feb 14;3(3):pgae069. doi: 10.1093/pnasnexus/pgae069 (PMC10904286; doi:10.1093/pnasnexus/pgae069)
Supplement: pgae069_Supplementary_Data [file pgae069_supplementary_data.pdf]

Supplementary Material for
Network Topology Facilitates Internet Traffic Control in Autocracies

Eda Keremoğlu *et al.*

*Corresponding author. E-mail: eda.keremoglu-waibler@uni-konstanz.de.

This PDF file includes:

Supporting Text
Figs. S1 to S5
Tables S1 to S11

1 Estimating Internet Traffic Flows

1.1 Access Providers

Determining which access providers are present in a country is a relatively straightforward process: only one autonomous system may own (or "originate") a block of IP address, known technically as an IP prefix. Further, in order to be globally reachable, access providers must disclose which IP ranges they own. To compute this information in each country, we use well-known and large-scale repositories of network prefixes (*1, 2*) combined with geographic information on each IP range (*3, 4*). This method is described in more detail in (*5*). Sampling bias is not an issue for our analyses of access provision as all countries are included in our sample.

1.2 Transit Providers

Our transit sample is built using a recently-developed methodology that identified 75 *transit-dominant* countries from all UN member states (*5*). These countries, in aggregate, represent approximately 1 billion users (or 26% of the world (*6*)). This methodology relies on large-scale network measurements and validation with telecommunications operators. The access networks in these countries rely primarily on transit providers for their connectivity to the global Internet. We exclude countries with access networks whose connectivity may rely primarily on bilateral *peering* agreements with foreign networks, as these proprietary agreements are difficult to observe in public data.

1.3 Country-Level Transit Influence

CTI Measure

Our quantification of transit influence relies on the Country-Level Transit Influence metric (CTI) (*5*). CTI, which ranges from 0 to 1, measures the potential capabilities that a given network (autonomous system, AS) has to observe or tamper with international traffic reaching users in a given country. In technical terms, CTI is based on graph theory and estimates the relative prevalence of a particular network on Internet routes serving a country. CTI relies on the assumption that networks with higher prevalence on Internet routes – more precisely, prevalence of the network on routes that reach a higher number of IP (v4) addresses and therefore users – are potentially privy to a higher share of the nation's traffic compared to networks with lower prevalence. This method processes existing data (*1, 2, 7*) on inter-operator connectivity in novel ways to mitigate bias; this connectivity information is extracted from Border Gateway Protocol (BGP) announcements.

CTI Validation and Stability

In the following, we expand on the supporting evidence for the validity of CTI's quantification of network influence based on the number of IP address they serve along the three directions introduced in the main paper. First, CTI's findings have been extensively validated with in-country experts from both the telecommunications industry and academia (*5*). From these discussions, CTI was found to be highly accurate at estimating which networks are most central to a country: in six countries, 90% of the networks inferred by CTI as being in the country's top 5 were confirmed by in-country experts; this finding is part of a broader discussion with in-country experts that engaged a total of "123 operators in 19 countries" (*5*). This larger effort provided substantial evidence supporting several key components of the model that we apply and that characterizes country-level networks (*5*) (CTI is the central metric in this model).

Second, since it is not feasible to collect traffic information at global scale, CTI characterizes how IP addresses are reached through transit providers, which is observable in public data (*8, 9*). We apply CTI on IPv4 addresses, which is a sufficient scope given that deployment of (newer protocol) IPv6 is not wide in Africa (*10, 11*), Latin America (*12*), and other developing regions (which comprise the vast majority of our sample countries). Since the model we apply has already established that transit providers are central to the connectivity of the countries in our sample (*5*), a fact which has also been validated with in-country experts, CTI is a reasonable estimate of network centrality that can be obtained from public data (and is therefore scalable). Further, CTI has been found to be stable both temporally and given changes in the IP-to-country database used as an input (*5*).

Third, IP addresses are a limited resource that is necessary to connect devices to the Internet. Previous work has found that networks serving a large number of IP addresses on BGP announcements (the data on inter-operator connectivity analyzed by CTI) also tend to carry larger volumes of traffic (*13*). Further, in these regions, IPv4 address allocation is constrained (*14*), which reduces the likelihood that IP addresses will be allocated and routed but unused.

1.4 Determining Ownership

We rely on a recently published worldwide list of state-owned Internet operators and their Autonomous System Numbers (ASNs), identifiers used to operate as ASs in the Internet network. The scale of the Internet network, with roughly 75,000 ASs as of October 2022, is the main obstacle to obtaining the ownership structure of each company. Therefore, we focus on the countries' most prominent Internet operators that satisfy the following conditions (i) of significant size, (ii) operating at a federal level (or equivalent), and (iii) who do not restrict their services to only certain sectors (*e.g.*, exclusively research and education).

Identifying state ownership of Internet operators operating as ASs on the network is a multidisciplinary process. This approach applies multiple stages of in-depth manual analysis of datasets that are highly diverse in nature that is fully described in (*15*). This process creates

lists of candidate ASs and candidate companies to then examine the ownership structure. The list of candidate ASs is composed of networks with significant footprints in countries' access markets holding at least 5% of countries' address space or the eyeballs. This list also includes countries' principal upstream providers identified by (*5*). The list of candidate companies contains telecommunication companies labeled as state-owned enterprises in Wikipedia articles and the Orbis database (*16*).

Entities in both lists of candidates are then validated examining the ownership structure of each company with authoritative data sources (e.g., companies' annual reports, world bank reports). These companies are finally added to the list if (i) the company has its own separate legal entity, (ii) the entity is partially controlled by a government unit, and (iii) the entity engages in commercial or economic activities. This work follows the definition given by the International Monetary Fund (*17*), which considers a firm as state-owned if the government owns at least 50% of its equity. There are additional organizations, such as universities, which are both controlled by the state and are also proprietors of IP addresses. However, since these organizations do not typically serve ordinary citizens directly (and therefore provide limited monitoring opportunities), we do not classify them as state-owned providers. Further details on these exclusions are described in (*15*).

To determine ownership of non-state providers, we assess their fractions of address space in a country. Among the primary purposes of an AS is the delivery of network traffic to its connected hosts, who are assigned an IP address by the AS. Therefore, the physical location of an AS's IP addresses is a direct indication of the AS's country of operation. We assign a provider to the country which is the maximum (in terms of IP addresses served) among all the countries in which this provider operates. We use an alternative threshold to assign non-state providers in the robustness checks below.

Overall, state-owned providers have considerable influence. For access provision, state providers service on average 28% of a country's address space. However, there is a large difference in where these providers come from: state providers from autocracies (operating domestically or abroad) control 37.4% of address space, whereas state providers based in democratic countries service on average 17.9% of a country's IP addresses. Transit providers owned by autocratic states exert a transit influence of 0.03 on average, whereas state-owned providers from democracies have a CTI of 0.02 (values for non-state providers are reported in Section 1.5).

As described above, we focus on prominent transit providers to determine ownership. We find that CTI quickly drops beyond the top providers in many countries. Based on these findings, we deem this approach to be appropriate. This is reflected in the low overall mean of AS-level CTI values and a global median of 0.00016 for state providers.

1.5 Descriptives

Table S1 presents mean values for access and transit service provision of state and non-state providers, further differentiating between the group of providers operating from all autocracies (including domestic country) and all democracies (including domestic country) operating in a given country. Mean values for transit service provision are given on the provider level. We find that, on average, the aggregate address space serviced by state providers is higher for the group of authoritarian owners (37.36%) than democratic ones (17.90%). Similarly, transit influence of authoritarian state providers (0.03) is higher than of providers owned by democratic governments (0.02).

	Access		Transit	
	State	Non-state	State	Non-state
All	28.38	59.80	0.03	0.01
Autocracy	37.36	49.28	0.03	0.01
Democracy	17.90	73.22	0.02	0.01

Table S1: Mean values of access service provision (fraction of address space) and transit influence (AS-level CTI) from state vs. non-state providers operating from foreign *and* domestic autocracies vs. foreign and domestic democracies.

2 Statistical Methodology

We estimate all our models using Ordinary Least Squares (OLS) regression with individual countries as our unit of observation. For our analyses of domestic influence, we aggregate the access and transit measures of influence at the country-level. We do this for each group of domestic providers, i.e. state and non-state, for each country. Since only one provider can serve as the access network for any IP address, to assess potential control at the access level, we take the sum of all IP addresses serviced by the respective provider group. This measure can range from 0% (if the provider group does not service any IP address) to 100% (if the provider group services all IP addresses in a country).

For country-level aggregations of transit influence, since multiple providers may indeed provide transit service to the same block of IP addresses, it is not possible to simply sum up their individual CTI values. Instead, we rely on a composite measure, CTIn, the pure-transit influence of the state; CTIn excludes IP address blocks where a state provider serves as the access network, since these were accounted for, and evaluates exactly once whether the state serves each IP block as a transit provider (regardless of how many state providers serve as transit networks). The CTIn measure can range from 0 to 1.

To analyse influence abroad, we assess the aggregate access and transit service provision of non-state and state providers operating from autocracies and democracies in countries abroad.

2.1 Variables

The regression models include the following variables.

Regime Type. Our main analyses rely on a binary regime type measure to assess topological differences between autocracies and democracies. To construct this measure, we use the Electoral Democracy Index (EDI) (variable "v2x_polyarchy") from (*18*) that ranges from 0 to 1, and assesses the degree to which states conform to an ideal model of an electoral democracy. Following (*19*), we take 0.5 as a threshold, classifying states with a EDI score below 0.5 as autocracies. We use alternative regime type indicators in the robustness tests below.

GDP per Capita. Our analyses control for (logged) GDP per capita (in current US$) from 2020, which we retrieve from (*20, 21*). This variable measures the level of development and wealth that may impact governments' capacity to act as a service provider and shape the network topology domestically and abroad.

Population. We consider the (logged) total size of a state's population in 2020 (*20, 21*). Topological differences may be linked to the number of citizens for whom service access and transit service has to be provided.

Internet Users. To proxy the degree of Internet penetration, our analyses control for the share of the population that uses the Internet (*20,21*). We use data from 2019 because it provides larger coverage than the data from 2020.

World Region. We account for regional effects by controlling for countries' continental location in Africa, Americas, Asia, Europe and Oceania based on the United Nation's official geographical classification (*22*). Europe is the reference world region in all our models.

3 Assessing Sampling Bias

Our analysis of transit influence relies on a sample of countries that, as described above, *heavily* rely on transit. This might challenge our results if this sampling strategy yields a bias in our sample of countries. This can be the case when a particular factor explains why some countries are heavily-reliant and, as a consequence, included in our sample. Geographic location, wealth or a particular institutional set-up are factors that can be linked to reliance on transit service provision. Table S2 assesses sampling bias by estimating the likelihood that any country (from the set of United Nations members) is included in our sample. Our dependent variable is a binary indicator of inclusion receiving a score of 1 if it is in our sample of transit analysis and 0 if is not. We run logistic regressions to assess whether a country's GDP p.c. (log), population size (log), share of population using the internet, geographical location and regime characteristics, measured with the continuous Electoral Democracy Index (Model 4) and our recoded binary regime type indicator (Model 5) increase its likelihood to be included in our sample. In our full models (4 and 5), a country's population size decreases its likelihood to be included in our transit sample. Neither the continuous (EDI) nor our recoded binary measure (Democracy)

are linked to sample inclusions (Model 4 and 5) respectively. This means that regime type, our main predictor, is not driving the selection of transit-reliant countries. Population size, on the other hand, is linked to sample inclusion in our full models, meaning that countries with larger populations are less likely to be part of our sample (odds ratio of 0.7). This, however, should not pose an issue for our analysis of topological differences between regime types as our focus lies on the particular role of state providers in autocracies vs. democracies. All our models control for population size to net out its influence.

	Inclusion in sample				
	(1)	(2)	(3)	(4)	(5)
GDP p.c. (log)	−0.31**	−0.30	−0.38	−0.11	−0.19
	(0.12)	(0.32)	(0.36)	(0.39)	(0.37)
Population (log)	−0.11	−0.10	−0.26	−0.34*	−0.36*
	(0.07)	(0.12)	(0.14)	(0.15)	(0.15)
Internet users		−0.01	−0.03	−0.04	−0.03
		(0.02)	(0.02)	(0.02)	(0.02)
Africa			−1.21	−1.48	−1.42
			(0.85)	(0.89)	(0.90)
Americas			0.94	0.97	1.16
			(0.73)	(0.75)	(0.76)
Asia			0.82	0.43	0.59
			(0.63)	(0.71)	(0.67)
EDI				−2.07	
				(1.17)	
Democracy (binary)					−1.01
					(0.52)
Constant	4.02*	4.02	8.60*	9.50*	9.60*
	(1.66)	(2.75)	(3.63)	(3.77)	(3.82)
Observations	184	124	124	123	123
Log Likelihood	−118.81	−75.04	−69.48	−67.30	−66.91
Akaike Inf. Crit.	243.61	158.08	152.96	150.60	149.83
Note:	*p<0.05; **p<0.01; ***p<0.001				

Table S2: Assessing sampling bias. Dependent variable: Inclusion in CTIn analysis sample. Logistic regressions. Reference world region: Europe.

4 Regression Results

This section presents the results of our main regression analyses, which the interaction figures in our article are based on. We start with models that include the interaction of our main predictors of interest only. We subsequently add controls for each country's *(i)* GDP p.c. and population size, *(ii)* share of the population using the internet, and *(iii)* geographical region. Our full models estimate the interaction effect between regime type and provider ownership on the extent of access and transit service provision while controlling for all covariates.

4.1 Access Provision by Domestic Providers

The models in Table S3 estimate how access provision by state and non-state providers differs by regime type. The result of Model 4 in Table S3 is visualized in Figure 1 (Panel B) in the main paper.

	Fraction of address space			
	(1)	(2)	(3)	(4)
Democracy	18.04***	18.94***	22.65***	20.31**
	(4.44)	(4.84)	(5.79)	(6.14)
State AS	−4.26	−6.47	−5.73	−5.48
	(4.93)	(5.06)	(6.14)	(6.17)
GDP p.c. (log)		0.66	−1.44	−1.80
		(1.37)	(3.42)	(3.65)
Population (log)		2.89**	2.93*	3.13*
		(1.07)	(1.30)	(1.40)
Internet users			0.08	0.11
			(0.18)	(0.19)
Africa				−1.93
				(8.25)
Americas				1.54
				(7.67)
Asia				−6.44
				(6.48)
Democracy x State AS	−34.31***	−32.22***	−37.72***	−37.88***
	(7.16)	(7.23)	(8.53)	(8.58)
Constant	44.98***	−7.61	4.01	5.53
	(3.26)	(21.40)	(28.67)	(35.90)
Observations	269	261	195	195
R^2	0.18	0.21	0.25	0.26
Adjusted R^2	0.18	0.20	0.23	0.22
Note:				*p<0.05; **p<0.01; ***p<0.001

Table S3: Fraction of address space serviced by all domestic state vs. non-state providers. Binary regime type measure recoded from continuous Electoral Democracy Index (*18*). Country-level OLS regressions. Reference world region: Europe.

4.2 Transit Service Provision by Domestic Providers

The models in Table S4 estimate how transit provision by state and non-state providers differs by regime type. The result of Model 4 in Table S4 is visualized in Figure 2 (Panel B) in the main paper.

	Country-level transit influence (CTIn)			
	(1)	(2)	(3)	(4)
Democracy	0.02	0.03	0.02	0.03
	(0.04)	(0.04)	(0.04)	(0.04)
State AS	0.14***	0.16***	0.18***	0.18***
	(0.04)	(0.04)	(0.04)	(0.04)
GDP p.c. (log)		−0.03*	−0.04	−0.03
		(0.01)	(0.02)	(0.03)
Population (log)		−0.01	−0.004	0.001
		(0.01)	(0.01)	(0.01)
Internet users			0.001	0.001
			(0.001)	(0.001)
Africa				0.03
				(0.07)
Americas				−0.03
				(0.06)
Asia				−0.003
				(0.05)
Democracy x State AS	−0.08	−0.09	−0.06	−0.06
	(0.06)	(0.06)	(0.06)	(0.06)
Constant	0.02	0.44	0.35	0.18
	(0.02)	(0.24)	(0.24)	(0.32)
Observations	93	89	62	62
R^2	0.16	0.21	0.35	0.37
Adjusted R^2	0.13	0.16	0.28	0.26
Note:				*p<0.05; **p<0.01; ***p<0.001

Table S4: Country-level transit influence of all domestic state vs. non-state providers. Binary regime type measure recoded from continuous Electoral Democracy Index (*18*). Reference world region: Europe.

4.3 Access Provision by Foreign Providers

The models in Table S5 estimate the extent to which international providers provide access in other countries, distinguishing between providers from democratic and autocratic countries ("providing") and between democratic and autocratic countries in which they operate ("receiving"). The result of Model 4 in Table S5 is visualized in Figure 3 (Panel B) in the main paper.

	Fraction of address space			
	(1)	(2)	(3)	(4)
Providing democracy	15.53***	14.80***	10.78***	10.78***
	(2.08)	(2.10)	(2.37)	(2.38)
Providing state AS	8.96***	9.99***	10.83***	10.83***
	(2.39)	(2.38)	(2.65)	(2.66)
Receiving democracy	−1.00	−0.99	−1.82	−1.84
	(2.03)	(2.10)	(2.30)	(2.39)
GDP p.c. (log)		−0.84	0.75	1.13
		(0.44)	(1.07)	(1.14)
Population (log)		−1.60***	−1.52***	−1.57***
		(0.35)	(0.38)	(0.41)
Internet users			−0.07	−0.07
			(0.06)	(0.06)
Africa				2.20
				(2.40)
Americas				2.76
				(2.22)
Asia				1.84
				(1.96)
Providing democracy x Providing state AS	−25.57***	−25.23***	−22.49***	−22.38***
	(3.42)	(3.44)	(3.89)	(3.90)
Providing democracy x Receiving democracy	1.15	1.87	6.05	5.97
	(2.87)	(2.84)	(3.11)	(3.13)
Providing state AS x Receiving democracy	−4.60	−4.46	−8.53*	−8.22*
	(3.33)	(3.28)	(3.52)	(3.56)
Prov. dem. x Prov. state AS x Receiving dem.	5.89	5.52	5.11	5.03
	(4.75)	(4.70)	(5.09)	(5.14)
Constant	2.16	35.10***	25.36**	21.28*
	(1.47)	(6.97)	(8.73)	(10.66)
Observations	538	521	396	392
R^2	0.23	0.26	0.27	0.27
Adjusted R^2	0.22	0.25	0.25	0.24

Note: *p<0.05; **p<0.01; ***p<0.001

Table S5: Fraction of address space serviced by all state vs. non-state providers from democratic and non-democratic countries in other democratic and non-democratic states. Reference world region: Europe.

5 Robustness Checks

5.1 Restricted Sample

We rerun our main analyses of access provision restricting our origin AS sample to those countries for which we have data at the transit level. Regression results for domestic access provision in Table S6, for access provision abroad (restriction based on receiving countries) in Table S7. Figure S1 plots respective interactions. The plots show that our results of domestic access (Panel A) are robust: state provision in autocracies does not differ from non-state provision whereas in democracies, state providers service significantly fewer users than non-state providers. The plot for access provision abroad (Panel B) shows that state providers from autocracies tend to provide access to a higher fraction in other autocracies than non-state providers, but this difference is not significant in our restricted sample. This can be explained by the higher uncertainty due to the reduced number of cases we analyse in these models.

	Fraction of address space
Democracy	19.72
	(10.21)
State AS	−15.49
	(9.48)
GDP p.c. (log)	−4.42
	(6.64)
Population (log)	1.75
	(2.90)
Internet users	0.26
	(0.30)
Africa	−3.12
	(15.87)
Americas	−4.53
	(14.66)
Asia	−9.05
	(12.64)
Democracy x State AS	−36.14*
	(15.76)
Constant	51.36
	(72.77)
Observations	66
R^2	0.29
Adjusted R^2	0.18
Note:	*p<0.05; **p<0.01; ***p<0.001

Table S6: Sample of origin AS analysis restricted to countries included in CTIn sample only. Restriction of receiving countries. Fraction of address space serviced by domestic state vs. non-state providers. Reference world region: Europe.

	Fraction of address space
Providing democracy	10.51**
	(3.40)
Providing state AS	12.98**
	(3.95)
Receiving democracy	−2.28
	(3.93)
GDP p.c. (log)	0.73
	(2.16)
Population (log)	−1.39
	(0.87)
Internet users	−0.05
	(0.09)
Africa	3.70
	(4.73)
Americas	7.95
	(4.28)
Asia	2.95
	(3.95)
Providing democracy x Providing state AS	−23.11***
	(5.53)
Providing democracy x Receiving democracy	2.07
	(5.26)
Providing state AS x Receiving democracy	−4.44
	(6.67)
Providing dem. x Providing state AS x Receiving dem.	4.38
	(9.49)
Constant	17.78
	(21.90)
Observations	131
R^2	0.23
Adjusted R^2	0.14
Note:	*p<0.05; **p<0.01; ***p<0.001

Table S7: Sample of origin AS analysis restricted to receiving countries included in CTIn sample only. Fraction of address space serviced by foreign state vs. non-state providers. Reference world region: Europe.

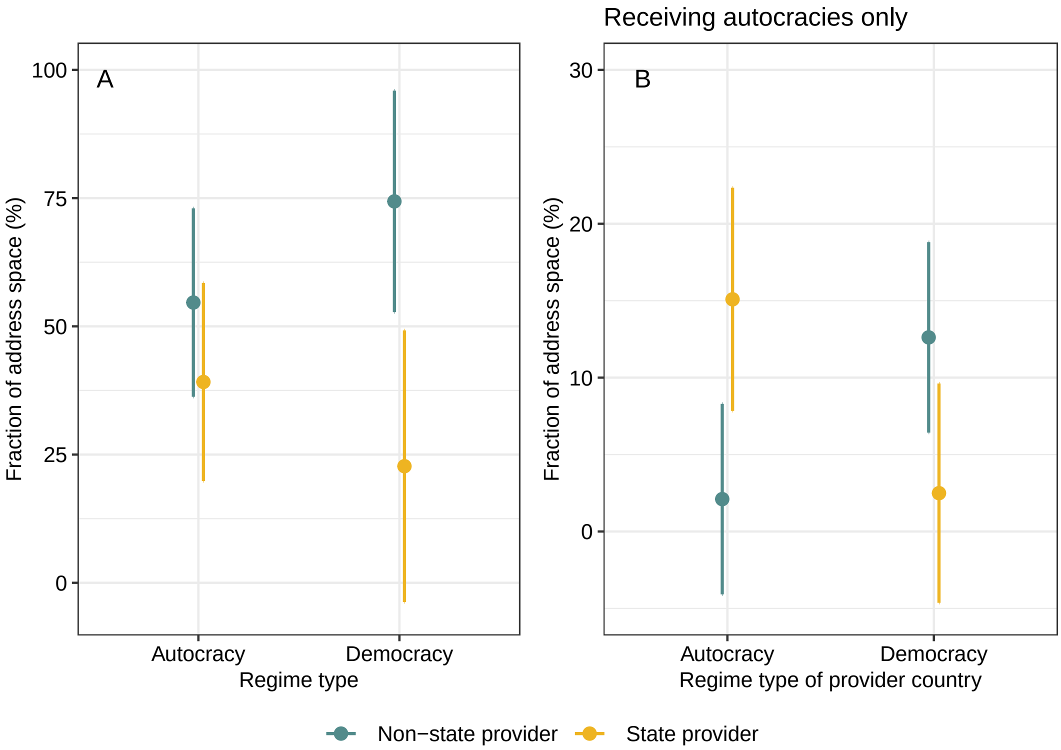

Figure S1: **A.** Fraction of address space serviced by all state (yellow) vs. non-state (green) domestic providers. Analysis run on reduced sample of access providers. Figure based on regression results from Table S6. **B.** Fraction of address space serviced by state vs. non-state providers in other autocracies only. Analysis run on reduced sample of access providers. Figure based on regression results from Table S7.

5.2 Alternative Regime Type Indicator

Our robustness checks present additional analyses using alternative regime type measures. First, we use the original, continuous measure of the Electoral Democracy Index (*18*) to rule out that recoding it to a dichotomous indicator impacts the results. Second, we rely on the Revised Combined Polity Score (Polity2) from (*23*). It is a composite index ranging from -10 (strongly autocratic) to +10 (strongly democratic) that assess states according to their recruitment of and constraints on the executive, and their openness of political participation. The indicator assesses democraticness in 2018, the latest year for which data is available. We assume that this lag of two years does not pose a challenge to our analysis. If any, only few cases, will see large changes in their Polity score, which leads us to presume that a country's score in 2018 will in most cases proxy its state in 2020. For our analysis of access provision abroad, we recode this score to a binary indicator. Countries receiving a score of 6 or above are coded as democracies, countries with a score below 6 are coded as non-democratic.

Table S8 shows regression results for domestic influence, Figure S2 plots their respective interactions. All plots confirm our main results. Access provision by state ASs does not differ from non-state ASs in autocracies when we use the continuous EDI (Panel A) or the Polity2 (Panel B), whereas state providers in democracies serve significantly fewer IP addresses than non-providers. Plots for transit influence also show that our findings remain robust when we use EDI (Panel C) or Polity2 (Panel D) as alternative measures for regime type. In autocracies, state providers are significantly more influential at the transit level than non-state providers, whereas this difference decreases the higher the level of democracy, and becomes insignificant in democratic states. Table S9 shows the results for access provision abroad. Figure S3 shows that state providers from autocracies tend to provide access to a higher fraction in other autocracies than non-state providers, but this difference is not significant when we use our alternative binary indicator, which we recode from Polity2.

	Fraction of address space		CTIn	
	(1)	(2)	(3)	(4)
EDI	47.37***		0.06	
	(13.10)		(0.09)	
Polity2		1.59**		0.0005
		(0.53)		(0.003)
State AS	17.92	−14.23**	0.25***	0.17***
	(9.38)	(5.13)	(0.07)	(0.03)
GDP p.c. (log)	−2.89	−1.15	−0.03	−0.03
	(3.75)	(3.70)	(0.03)	(0.03)
Population (log)	3.13*	2.70	−0.002	−0.003
	(1.37)	(1.46)	(0.01)	(0.01)
Internet users	0.14	0.14	0.001	0.0004
	(0.19)	(0.20)	(0.001)	(0.001)
Africa	−0.72	1.23	0.02	0.02
	(8.20)	(8.63)	(0.07)	(0.07)
Americas	2.66	4.17	−0.03	−0.02
	(7.52)	(7.65)	(0.06)	(0.06)
Asia	−5.06	−3.97	−0.01	−0.02
	(6.80)	(7.10)	(0.05)	(0.05)
EDI x State AS	−82.95***		−0.23	
	(16.18)		(0.13)	
Polity2 x State AS		−3.02***		−0.01*
		(0.67)		(0.004)
Constant	−2.00	6.95	0.21	0.27
	(35.36)	(36.69)	(0.32)	(0.32)
Observations	195	185	62	59
R^2	0.28	0.28	0.39	0.43
Adjusted R^2	0.25	0.24	0.28	0.33
Note:		*p<0.05; **p<0.01; ***p<0.001		

Table S8: Main analyses of domestic influence using alternative regime type measures. DV Models 1-2: Percentage of address space, Models 3-4: Country-level transit influence. Reference world region: Europe.

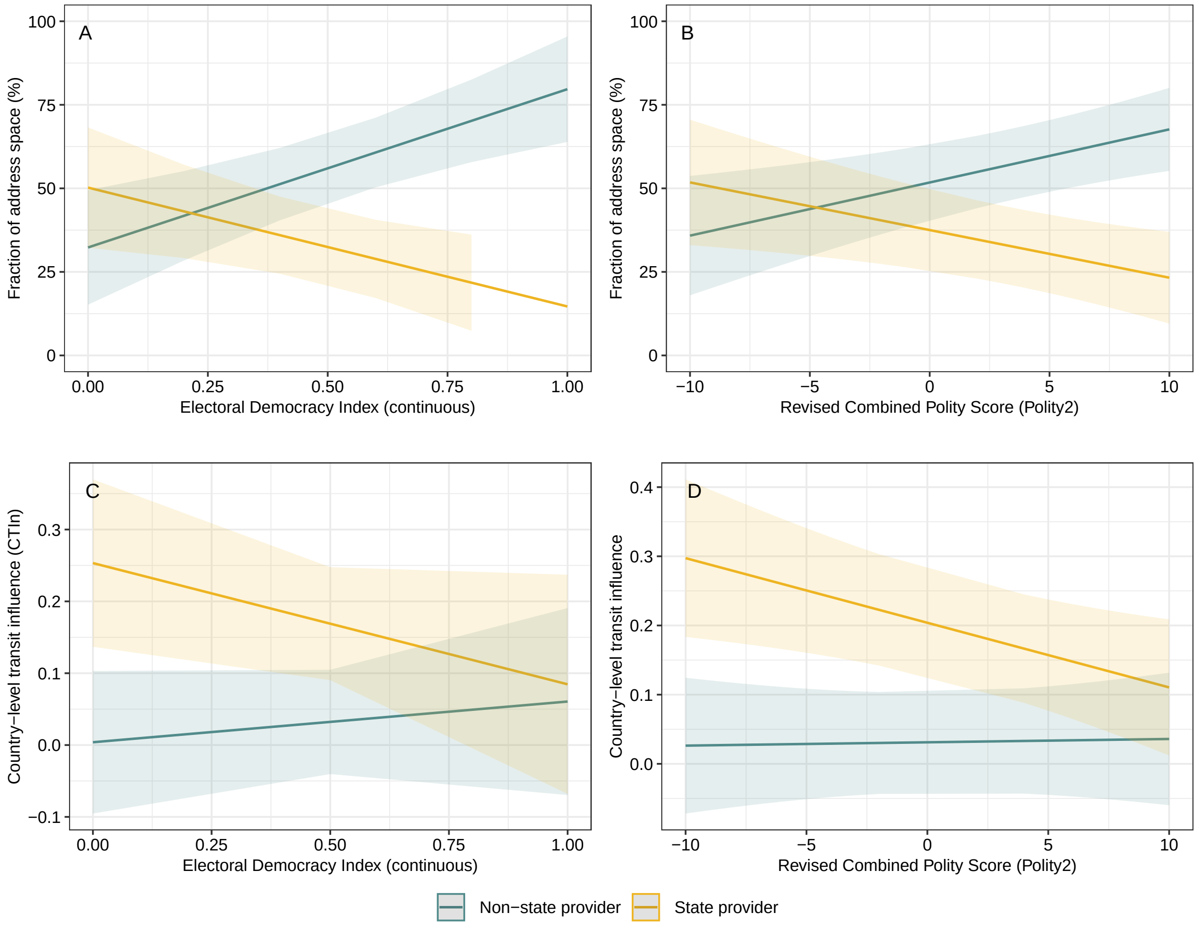

Figure S2: **A.** Fraction of address space serviced by all state (yellow) vs. all non-state (green) domestic providers using the continuous Electoral Democracy Index (*18*) as an alternative regime type measure. Figure based on regression results in Table S8, Model 1. **B.** Fraction of address space serviced by all state (yellow) vs. all non-state (green) domestic providers using the Revised Combined Polity Score (*23*) as an alternative regime type measure. Figure based on regression results in Model 2. **C.** Country-level transit influence of all state (yellow) vs. non-state (green) domestic providers using the continuous Electoral Democracy Index (*18*) as an alternative regime type measure. Figure based on regression results in Model 3. **D.** Country-level transit influence of all state (yellow) vs. non-state (green) domestic providers using the Revised Combined Polity Score (*23*) as an alternative regime type measure. Figure based on regression results in Model 4.

.

	Fraction of address space
Providing democracy	10.74***
	(2.81)
Providing state AS	8.97**
	(3.18)
Democracy	−0.98
	(2.72)
GDP p.c. (log)	1.54
	(1.17)
Population (log)	−1.46**
	(0.45)
Internet users	−0.08
	(0.06)
Africa	2.42
	(2.58)
Americas	2.53
	(2.25)
Asia	2.25
	(1.95)
Providing democracy x Providing state AS	−20.86***
	(4.49)
Providing democracy x Receiving democracy	5.10
	(3.45)
Providing state AS x Receiving democracy	−7.65
	(3.95)
Providing dem. x Providing state AS x Receiving dem.	4.88
	(5.51)
Constant	16.61
	(11.28)
Observations	355
R^2	0.27
Adjusted R^2	0.24
Note:	*p<0.05; **p<0.01; ***p<0.001

Table S9: Fraction of address space serviced by state vs. non-state providers from democracies and non-democracies in other democracies and non-democracies. Binary regime type indicator recoded from Revised Combined Polity Score (*23*) (autocracy if Polity2 <6). Reference world region: Europe.

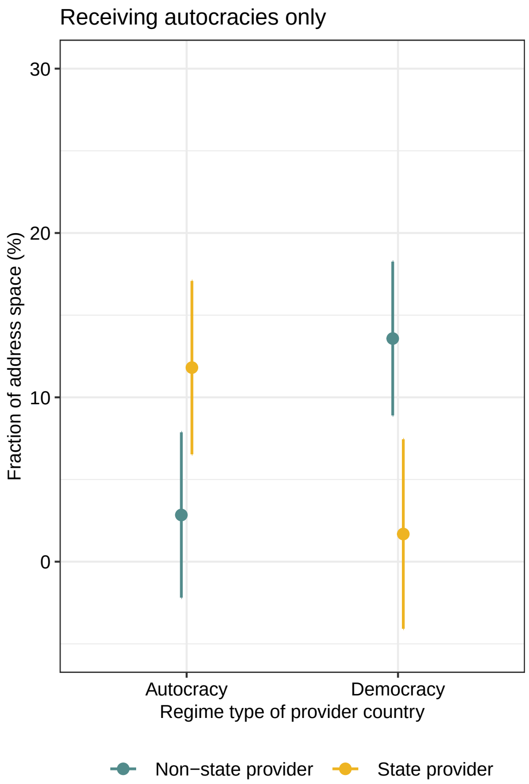

Figure S3: Fraction of address space serviced by state (yellow) vs. non-state (green) providers in other autocracies only. Binary regime type indicator recoded from Revised Combined Polity Score (autocracy if Polity2 <6) (*23*).

5.3 Alternative Assignment of ASs to Countries

We rerun our main analyses using an alternative approach to determine ownership of ASs. We assign a non-state AS to that country where it services at least two thirds of its addresses. This does not fundamentally alter the nationality assignment of non-state ASs. Rather, it reduces our sample as ASs that do not reach the threshold of two thirds of IP addresses cannot be assigned and are excluded from the sample (9731 access ASs, 2908 transit ASs).

Table S10 shows regression results for access provision and transit influence of domestic providers. Figure S4 confirms our main findings of access provision (Panel A). At the transit level (Panel B) an alternative assignment of ASs does not show a significant difference between state and non-state providers in autocracies. This, however, is most likely due to the high number of non-state ASs we exclude. As only ASs that reach a threshold of two thirds of IP address remain in our sample, it will over-represent those that have a higher influence over address space. Results for access provision abroad are reported in Table S11. Figure S5 confirms our main results.

	Fraction of address space	CTIn
	(1)	(2)
Democracy	17.89**	0.08
	(6.09)	(0.06)
State AS	−2.98	0.17**
	(6.11)	(0.05)
GDP p.c. (log)	−2.42	−0.04
	(3.62)	(0.03)
Population (log)	2.73	0.01
	(1.38)	(0.02)
Internet users	0.11	0.002
	(0.19)	(0.002)
Africa	−1.52	0.08
	(8.18)	(0.09)
Americas	0.88	−0.07
	(7.60)	(0.08)
Asia	−6.30	−0.01
	(6.42)	(0.06)
Democracy x State AS	−34.97***	−0.09
	(8.51)	(0.08)
Constant	14.85	0.13
	(35.58)	(0.37)
Observations	195	47
R^2	0.21	0.39
Adjusted R^2	0.17	0.25
Note:	*p<0.05; **p<0.01; ***p<0.001	

Table S10: Alternative AS nationality assignment. Fraction of address space originated (Model 1) and transit influence (Model 2) of domestic state vs. non-state providers. Reference world region: Europe.

	Fraction of address space
Providing democracy	0.39
	(0.36)
Providing state AS	5.27***
	(0.51)
Receiving democracy	0.05
	(0.41)
GDP p.c. (log)	−0.28*
	(0.14)
Population (log)	−0.29***
	(0.05)
Internet users	−0.003
	(0.01)
Africa	0.17
	(0.31)
Americas	0.36
	(0.24)
Asia	0.05
	(0.24)
Providing democracy x Providing state AS	−5.70***
	(0.76)
Providing democracy x Receiving democracy	−0.13
	(0.43)
Providing state AS x Receiving democracy	−4.46***
	(0.63)
Providing dem. x Providing state AS x Receiving dem.	5.59***
	(0.92)
Constant	7.94***
	(1.29)
Observations	2,317
R^2	0.09
Adjusted R^2	0.09
Note:	*p<0.05; **p<0.01; ***p<0.001

Table S11: Alternative AS nationality assignment. Fraction of address space originated (Model 1) and transit influence (Model 2) of state vs. non-state providers abroad. Reference world region: Europe.

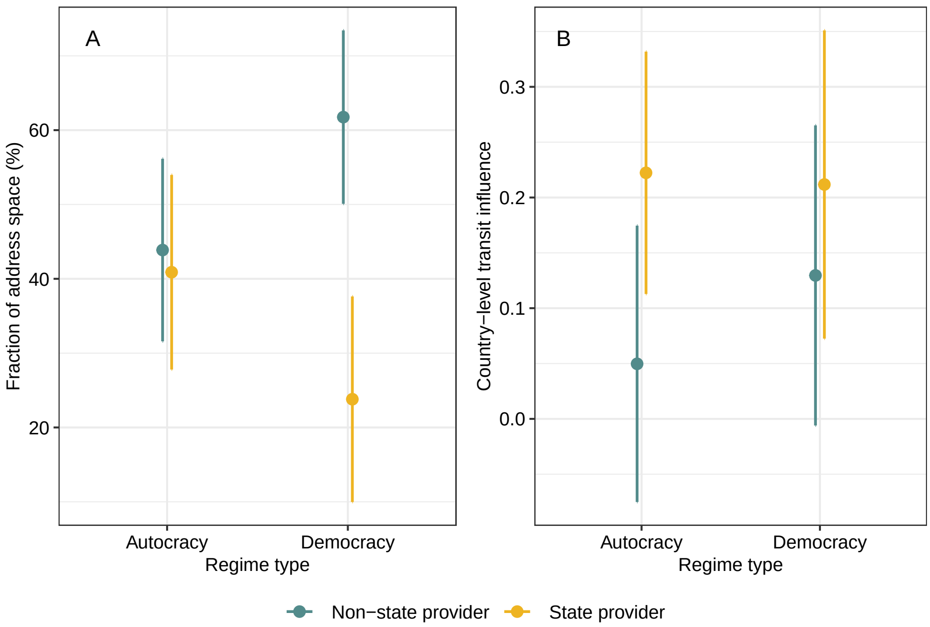

Figure S4: **A.** Fraction of address space serviced by state (yellow) vs. non-state (green) domestic providers using two thirds of IP addresses as a threshold to assign non-state providers to countries. **B.** Country-level transit influence of state (yellow) vs. non-state (green) domestic providers using two thirds of IP addresses as a threshold to assign non-state providers to countries.

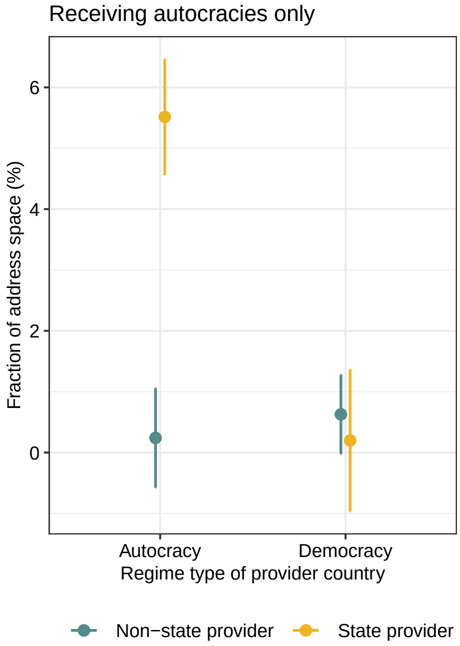

Figure S5: Fraction of address space serviced by state (yellow) vs. non-state (green) providers in democracies and non-democracies in other democracies and non-democracies using two thirds of IP addresses as a threshold to assign non-state providers to countries.

References

1. RouteViews (2019). Available at http://www.routeviews.org/routeviews/.

2. RIPE Routing Information Service (RIS) (2019). Available at https://www.ripe.net/analyse/internet-measurements/routing-information-service-ris.

3. Netacuity (2020). Available at http://info.digitalelement.com/.

4. RIR Delegation Files (2019). Available at https://ftp.ripe.net/pub/stats/ripencc/.

5. A. Gamero-Garrido, *et al.*, *International Conference on Passive and Active Network Measurement* (Springer, 2022), pp. 645–674.

6. The World Factbook. Available at https://www.cia.gov/the-world-factbook/field/internet-users/country-comparison.

7. CAIDA AS-Relationships (2019). Available at http://data.caida.org/datasets/as-relationships/.

8. M. Luckie, B. Huffaker, A. Dhamdhere, V. Giotsas, K. Claffy, *ACM IMC* (2013).

9. A. Dhamdhere, C. Dovrolis, *ACM Internet Measurement Conference (IMC)* (2008).

10. E. Agbaraji, F. Opara, M. Aririguzo, *IJAET* **2**, 315 (2012).

11. I. Livadariu, A. Elmokashfi, A. Dhamdhere, *e-Infrastructure and e-Services for Developing Countries*, V. Odumuyiwa, O. Adegboyega, C. Uwadia, eds. (Springer International Publishing, Cham, 2018), pp. 345–351.

12. S. D. P. Affairs, Statistics and trends: Ipv6 deployment in latin america and the caribbean 2016-2020 (2021). Available at https://www.lacnic.net/innovaportal/file/3052/1/estadisticas-y-tendencias-el-despliegue-de-ipv6-en-lac-2016-2020-en.pdf.

13. A. Lodhi, N. Larson, A. Dhamdhere, C. Dovrolis, k. claffy, *ACM CCR* (2014).

14. A. Dainotti, *et al.*, *IEEE Journal on Selected Areas in Communications* **34**, 1862 (2016).

15. E. Carisimo, A. Gamero-Garrido, A. C. Snoeren, A. Dainotti, *Proceedings of the 21st ACM Internet Measurement Conference*, IMC '21 (Association for Computing Machinery, New York, NY, USA, 2021), pp. 687–702.

16. B. van Dijk, Orbis Overview (2014). Available at https://www.bvdinfo.com/en-us/our-products/data/international/orbis.

17. International Monetary Fund, State-owned enterprises: The Other Government (2020). Available at https://www.imf.org/~/media/Files/Publications/fiscal-monitor/2020/April/English/ch3.ashx.

18. M. Coppedge, *et al.*, *V-Dem [Country–Year/Country–Date] Dataset v12*, Varieties of Democracy (V-Dem) Project, https://doi.org/10.23696/vdemds22 (2022).

19. A. Lührmann, M. Tannenberg, S. I. Lindberg, *Politics and Governance* **6**, 60 (2018).

20. D. Kaufmann, A. Kraay, M. Mastruzzi, *Hague Journal on the Rule of Law* **3**, 220 (2011).

21. World Bank, World Development Indicators. Available at https://data.worldbank.org.

22. United Nations Statistics Division, Standard country or area codes for statistical use (m49) (2022). Available at https://unstats.un.org/unsd/methodology/m49.

23. M. G. Marshall, T. R. Gurr, *Polity 5. Political Regime Characteristics and Transitions, 1800-2018. Dataset Users' Manual*, Center for Systemic Peace (2020).
